# Dynamics of the genetic diversity of oat varieties in the Tyumen region at avenin-coding loci

**DOI:** 10.18699/VJ20.607

**Published:** 2020-03

**Authors:** A.V. Lyubimova, G.V. Tobolova, D.I. Eremin, I.G. Loskutov

**Affiliations:** Scientific Research Institute of Agriculture of the Northern Trans-Ural Region – Branch of the Tyumen Scientific Center of Siberian Branch of the Russian Academy of Sciences, Moskowsky village, Tyumen district, Tyumen region, Russia Northern Trans-Ural State Agricultural University, Tyumen, Russia; Northern Trans-Ural State Agricultural University, Tyumen, Russia; Northern Trans-Ural State Agricultural University, Tyumen, Russia; Federal Research Center the N.I. Vavilov All-Russian Institute of Plant Genetic Resources (VIR), St. Petersburg, Russia

**Keywords:** oat, variety, electrophoresis, storage proteins, avenin, avenin-coding loci, alleles, genetic diversity, овес, сорт, электрофорез, запасные белки, авенин, авенин-кодирующие локусы, аллель, генетическое разнообразие

## Abstract

Molecular and biochemical markers are used to analyze the intraspecific genetic diversity of crops. Prolamin-coding loci are highly effective for assessing this indicator. On the basis of the Laboratory of Varietal Seed Identification of the State Agrarian University of the Northern Trans-Urals, 18 varieties of common oat included in the State Register of Selection Achievements in the Tyumen Region from the 1930s to 2019 were studied by electrophoresis in 2018–2019. The aim of the work was to study the dynamics of the genetic diversity of oat varieties at avenin-coding loci. For the analysis, 100 grains of each variety were used. Electrophoresis was carried out in vertical plates of 13.2 % polyacrylamide gel at a constant voltage of 500 V for 4.0–4.5 h. It was found that 44.4 % of the varieties are heterogeneous, each consisting of two biotypes. For three loci, 20 alleles were identified, 10 of which were detected for the first time. The allele frequency of avenin-coding loci varied with time. In the process of variety exchange, alleles that are characteristic of varieties of non-Russian origin were replaced by alleles present in domestic varieties and then in the varieties developed by local breeding institutions. The following alleles had the highest frequency in Tyumen varieties: Avn A4 (50.0 %), A2 (25.0 %), Avn B4 (50.0 %), Bnew6 (37.5 %), Avn C1 (37.5 %), C2 and C5 (25.0 %). These alleles are of great value as markers of agronomically and adaptively important characters for the region in question. The amount of genetic diversity of oats varied with time from 0.33 in 1929–1950 to up to 0.75 in 2019. The high value of genetic diversity in modern breeding varieties of the Scientific Research Institute of Agriculture of the Northern Trans-Urals and an increase in this indicator over the past 20 years are associated with the use of genetically heterogeneous source material in the breeding process. This allowed obtaining varieties with high adaptive potentials in the natural climatic conditions of the region.

## Abstract (ru)

Для анализа внутривидового генетического разнообразия сельскохозяйственных культур применяются разнообразные молекулярные и биохимические маркеры. Высокой эффективностью при оценке этого показателя обладают проламин-кодирующие локусы. На базе лаборатории сортовой идентификации семян Государственного аграрного университета Северного Зауралья в 2018–2019 гг. методом электрофореза исследованы 18 сортов овса посевного, включенных в Государственный реестр селекци- онных достижений по Тюменской области с 1930-х гг. до 2019 г. Целью работы было изучить динамику генетического разнообразия сортов по авенин-кодирующим локусам. Для анализа использовали по 100 зерновок каждого сорта. Электрофорез проводили в вертикальных пластинах 13.2 % полиакриламидного геля при постоянном напряжении 500 В в течение 4.0–4.5 ч. Установлено, что 44.4 % сортов гетерогенны и состоят из двух биотипов. Для трех локусов идентифицировано 20 аллелей, 10 из которых выявлены впервые. Частота встречаемости аллелей авенин-кодирующих локусов изменялась с течением времени. Аллели, характерные для сортов иностранного происхождения, в процессе сортосмены заместились аллелями, присутствующими в отечественных сортах, а затем в сортах местных селекционных учреждений. Наибольшую частоту встречаемости в сортах тюменской селекции имели аллели Avn A4 (50.0 %), A2 (25.0 %), Avn B4 (50.0 %), Bnew6 (37.5 %), Avn C1 (37.5 %), C2 и C5 (25.0 %). Эти аллели имеют большую ценность как маркеры хозяйственно ценных и адаптивно значимых признаков. Величина генетического разнообразия в сортах овса изменялась с течением времени от 0.33 в 1929–1950 гг. до 0.75 в 2019 г. Высокое значение генетического разнообразия в современных сортах селекции Научно-исследовательского института сельского хозяйства Северного Зауралья, а также увеличение этого показателя на протяжении последних 20 лет связаны с использованием в селекционном процессе генетически разнородного исход- ного материала. Это позволило получить сорта, обладающие высоким адаптивным потенциалом в при- родно-климатических условиях данного региона.

## Introduction

Common oat (Avena sativa L.) is a valuable agricultural crop used both for food and animal feed (Barsila, 2018). An important factor in increasing the production of oat is the creation of new intensive type varieties characterized by high productivity and environmental sustainability (Goncharenko, 2016). In the Tyumen region, breeding work with this culture is very active. From the first half of the twentieth century to the present, 18 varieties of spring oat have been included in the State Register of Selection Achievements in the region. In 1993, the first variety of local breeding, Megion, was regionalized. The proportion of varieties created by the Scientific Research Institute of Agriculture of the Northern Trans-Urals in the region’s crops has since been constantly increasing. Nowadays, only varieties of local breeding are included in the State Register of Selection Achievements in the region.

However, active breeding can lead to a decrease in the genetic diversity of the species. This is due to the frequent involvement of the same genotypes in the breeding process to enhance specific agronomic characters. A decrease in genetic diversity negatively affects the resistance of populations to diseases and the populations’ ability to adapt to changing environmental and climatic conditions (Novoselskaya-Dragovich et al., 2007; Afanasenko, Novozhilov, 2009; Goncharenko, 2016).

A variety of molecular and biochemical markers are used to analyze intraspecific genetic diversity (Konarev et al., 2000; Montilla-Bascón et al., 2013; Shavrukov, 2016; Scheben et al., 2017). Prolamin-coding loci are very effective for assessing this indicator (Che, Li, 2007; Melnikova et al., 2010; Kudryavtsev et al., 2014; Lyalina et al., 2016; Lyubimova, Eremin, 2018a; Zobova et al., 2018; Utebayev et al., 2019). Prolamins of oat (avenins) are inherited as blocks and are controlled by three independent loci: Avn A, Avn B and Avn C, located in three homeologous chromosomes of group A (Portyanko et al., 1987, 1998). Due to the high level of avenin polymorphism, almost every oat variety, biotype, or line is characterized by a unique component composition of storage proteins (Loscutov, 2007; Lyubimova, Eremin, 2018b). This allows analyzing the individual allele frequency of avenin-coding loci, the dynamics of changes in their occurrence in time and space, and also assessing the genetic transformations that occur under the influence of prolonged artificial selection.

The aim of the work is to study the dynamics of genetic diversity at avenin-coding loci in common oat varieties included in the State Register of Selection Achievements in the Tyumen region from the 1930s to the present for assessing the effectiveness of selection work carried out in the region.

## Materials and methods

The studies were carried out in the Laboratory of Varietal Seed Identification of the Agrobiotechnological Center of the Northern Trans-Urals State Agrarian University in 2018–2019. Eighteen varieties of common oat included in the State Register of Selection Achievements in the Tyumen Region since 1929 were studied (Table 1).

**Table 1.**
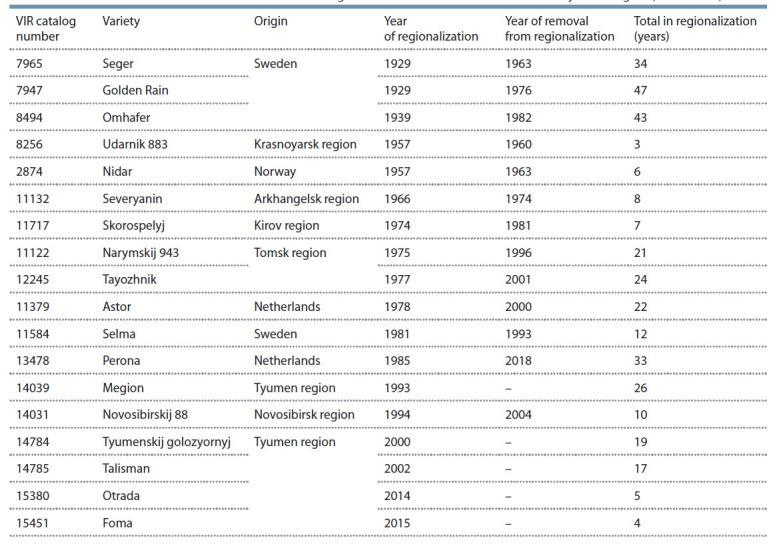
Varieties of common oat included in the State Register of Selection Achievements in the Tyumen region (1929–2019)

Plant material was provided from the collection of the Federal Research Center N.I. Vavilov All-Russian Institute of Plant Genetic Resources and the institution-originator of varieties, the Scientific Research Institute of Agriculture of the Northern Trans-Urals, a Branch of the Tyumen Scientific Center of Siberian Branch of the Russian Academy of Sciences.

For laboratory analysis, 100 grains of each variety selected by random sampling were used. For one-dimensional electrophoresis of avenins, a published technique (Portyanko et al., 1998) with modifications was used. Proteins were extracted from individual crushed grains by adding 90 μl of 70 % ethanol. The obtained extract was centrifuged, and 300 μl of methylene green dye was added to it. Protein extract (22 μl) was added to the polyacrylamide gel. Gel composition: 13.17 g of acrylamide, 0.66 g of N,Nʹ-methylenebis- acrylamide, 7.17 g of urea, 2.0 mg of iron sulfate (III), 80.0 mg of ascorbic acid, and 0.26 g of aluminum lactate. All reagents were dissolved in 100 ml aluminum-lactate buffer (pH 3.1). Acrylamide polymerization was initiated by adding 25 μl of 15 % hydrogen peroxide to 75 ml of a gel solution. Electrophoresis was carried out in vertical electrophoretic chambers with dimensions of the formed plates of 17.8 × 17.8 × 0.15 cm (VE-20, Helicon, Russia) for 4.0–4.5 h at a constant voltage of 500 V. To fix and stain the gel, a 10 % solution of trichloroacetic acid with the addition of 0.05 % Coomassie brilliant blue R-250 in ethanol was used. Identification of allelic variants of component blocks controlled by avenin-coding loci was carried out on the basis of a catalog developed by V.A. Portyanko et al. (1987). Astor common oat (Avn A2 B4 C2) were used as a standard. In case the detected block was not in the catalog, it was marked with a “new” mark.

In order to assess the dynamics of the change in the genetic diversity of oat varieties over time, all the studied samples were grouped. One group included varieties cultivated in the same ten-year period. The gene diversity at the locus (H) was calculated for each group of varieties separately according to the following formula:

**Formula:**
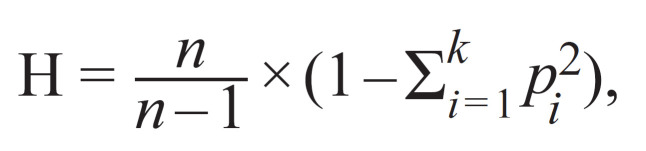
1.

where p_i_ is the population frequency of the i-th allele; k is the number of locus alleles; n is the sample size (Nei, 1987). To calculate the average gene diversity (H), the number of alleles per locus was averaged over all loci. The calculations were performed using the Arlequin Ver 3.5.2.2 program (Copyright 2015 L. Excoffier. CMPG, University of Berne).

## Results

As a result of the studies, it was found that 8 (44.4 %) of the 18 analyzed varieties were heterogeneous in the composition of avenin. Seger, Golden Rain, Omhafer, Severyanin, Narymskij 943, Tayozhnik, Megion and Otrada varieties consisted of two biotypes. These varieties are characterized by the presence of several alleles at one or more avenincoding loci. In the genetic formula, such states of loci were recorded with the “+” sign (Table 2). In subsequent calculations, each biotype was considered by us as a separate sample. A total of 26 samples were examined.

**Table 2.**
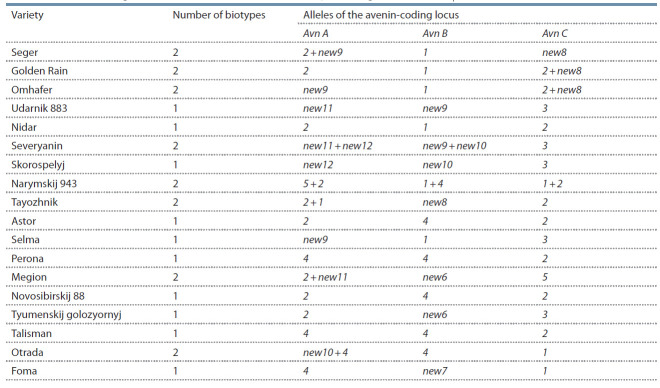
Alleles of avenin-coding loci of common oat varieties included in the State Register of Selection Achievements in the Tyumen region (1929–2019)

An analysis of the electrophoretic spectra of avenin allowed us to describe the genetic formulas for each of the studied varieties. Altogether, 8 alleles were detected for the Avn A locus; 7, for the Avn B locus; and 5, for the Avn C locus. It should be noted that some of the combinations of avenin components that we found were absent in the catalog of genetic nomenclature. To identify new blocks of components, it is necessary to conduct a hybridological analysis and assess the nature of the inheritance of avenin components. However, we highlighted the alleged blocks of components, each of which was assigned a number following the blocks previously described in the catalog. A “new” mark was added before the number of each of the proposed blocks.

To assess genetic diversity at different time intervals, we calculated the allele frequency of avenin-coding loci (Table 3).

**Table 3.**
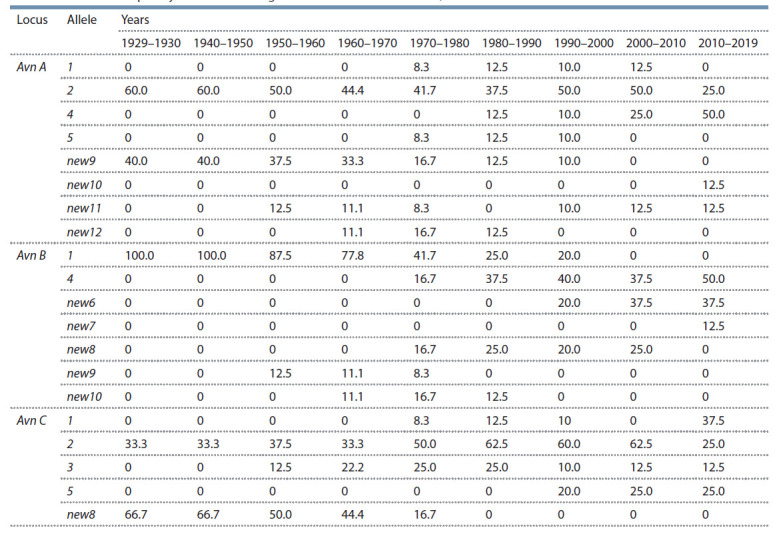
The allele frequency of avenin-coding loci of common oat varieties, %

**Fig. 1.**
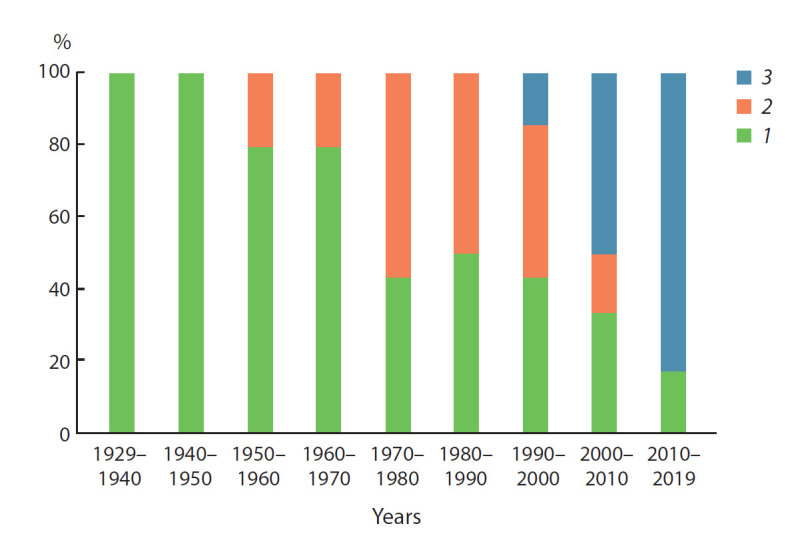
The dynamics of the regionalized assortment of common oat in the Tyumen region (1929–2019). Varieties: 1 – of foreign breeding; 2 – of domestic breeding; 3 – of local breeding institutions.

Different alleles predominate in different groups of varieties. For the Avn A locus, only alleles 2 and new9 were found before 1950. However, the frequency of their occurrence began to decrease with the appearance of domestic varieties and then varieties of local breeding in the crops of the region (Fig. 1). Alleles 1, 5 and new12 were characteristic of varieties cultivated from 1960 to 2010, and are no longer found today. Allele 4 (50.0 %) is currently the most widespread; allele 2 accounts for 25.0 %; new11 and new12, for 12.5 % each.

For the Avn B locus of modern oat varieties, alleles 4 (50.0 %) and new6 (37.5 %) predominate; new7 is found with a frequency of 12.5 %. Alleles 1, new8, new9 and new10, which are characteristic of varieties of foreign and domestic breeding, but not found by us among the varieties of local breeding, are completely eliminated.

A similar situation is observed for the Avn C locus: allele new8, which occurred with a frequency of 66.7 % in 1929–1950, is currently replaced by alleles 1 (37.5 %), 5 (25.0 %) and 3 (12.5 %). It is necessary to pay attention to allele 2, the presence of which in varieties has been noted at all periods of cultivation ever since 1929. This allele frequency ranged from 25.0 to 62.5 %. Nowadays, this allele is presented in 25.0 % of the varieties. The same feature was noted for allele 2 of the Avn A locus.

**Fig. 2.**
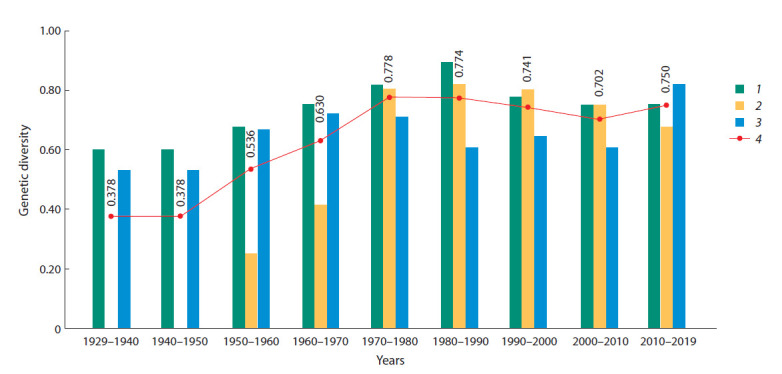
Genetic diversity of oat varieties by avenin-coding loci. Locuses: 1 – Avn A; 2 – Avn B; 3 – Avn C; 4 – average gene diversity.

The value of genetic diversity, calculated on the basis of data on the allele frequency, also changed over time (Fig. 2).

This indicator was minimal before 1950 (0.38), when only three varieties of oat were cultivated in the region: Seger, Golden Rain and Omhafer. Subsequently, with the advent of new varieties in the region’s crops, the value of genetic diversity increased, reaching its maximum in the period from 1970 to 1980 (0.78). During this period of time, an active variety exchange was carried out in the region – Seger, Udarnik 883 and Nidar were removed from regionalization, and they were replaced by Skorospelyj, Narymskij 943, Tayozhnik and Astor varieties bearing new alleles of avenin-coding loci. The period 1970–1980 was characterized by the largest variety of allelic variants in varieties – 15 alleles were found at three Avn loci (Table 3). Subsequently, in the process of replacing foreign varieties with domestic ones, the indicator of genetic diversity decreased to 0.70 by 2010. A decrease in diversity was caused by the exclusion from regionalization of a large number of varieties bearing alleles not found in varieties of local breeding. However, to date, there has been an increase in average gene diversity to 0.75.

## Discussion

As a result of our analysis using multiple alleles of avenincoding loci, we described the genetic formulas for 18 va- rieties of common oat included in the State Register of Selection Achievements in the Tyumen region. It was established that the heterogeneity of varieties is 44.4 %. The presence of several biotypes increases the adaptive potential of the variety (Metakovsky, 1990; Novoselskaya- Dragovich et al., 2013), which is extremely important in the natural climatic conditions of the Tyumen region, which is a risky farming zone.

In some varieties, identical prolamin spectra were found. Thus, the first and second biotypes of Golden Rain are identical to Seger (I biotype) (2.1.new8) and Nidar (2.1.2), respectively. The second biotype of Seger coincided with the second biotype of Omhafer (new9.1.new8). The first and second biotypes of Severyanin coincide with Udarnik (new11.new9.3) and Skorospelyj (new12.new10.3). The same types of spectra are characteristic of the second biotype of Narymskij 943 as well as Astor and Novosibirskij 88 (2.4.2); the spectra of Perona and Talisman (4.4.2) coincide. As a result of the analysis, it was found that only 10 (38.5 %) of the 26 studied genotypes are variety-specific. This is a fairly low rate.

The identity of alleles of prolamin-coding loci in varieties is associated with the involvement of the same genotypes in breeding programs (Portyanko et al., 1998; Melnikova et al., 2010; Novoselskaya-Dragovich et al., 2013). For example, Seger and Golden Rain were bred from the same oat variety Milton (=Propsteier), and Omhafer, too, originated from it. The old oat variety Milton appeared in northern Germany and was widespread in northern Europe (Portyanko et al., 1998). Apparently, this variety possessed outstanding economic characteristics, which led to its frequent inclusion in the breeding process. This was reflected in the matching set of alleles of avenin-coding loci in its descendants. The presence of varieties with the same genetic formulas of prolamins reduces the efficiency of using the method of electrophoresis for their differentiation. A number of authors in their studies concluded that the use of avenin-coding loci as the only marker system for distinguishing a large number of oat varieties is insufficient, since the allelic diversity of oat prolamin loci is characterized as low compared to wheat, barley and rye (Cliff, Cooke, 1984; Souza, Sorrels, 1990; Portyanko et al., 1998). In such cases, there is a need for an additional use of other marker systems (Wight et al., 2010). However, it should be noted that the modern varieties of oat created by the Scientific Research Institute of Agriculture of the Northern Trans-Urals have an individual allelic composition of avenin-encoding loci, which makes it possible to differentiate their genotypes with high accuracy.

An analysis of the frequencies of alleles of avenin-coding loci for all three loci allowed us to note the relationship between the frequency of alleles and a set of cultivated varieties, especially their origin. In the process of variety exchange, the alleles characteristic of varieties of foreign breeding were gradually replaced by alleles present in domestic varieties, and then in the varieties developed of local breeding istitutions. A similar replacement of one allele with another during breeding work was noted by many researchers in the study of prolamin-coding loci of wheat and barley (Novoselskaya-Dragovich et al., 2007; Lyalina et al., 2016). On a large number of examples, the adaptive nature of prolamin polymorphism has been proved. Their connection with adaptive gene complexes allows, based on the spectra of storage proteins, identifying genotypes that are most adapted to specific climatic conditions. A.Yu. Novoselskaya-Dragovich and the co-workers (2013) noted that genetic differences between varieties of different geographical origin are determined by natural selection. In this case, the reason for the rather rapid replacement of the “old” alleles with “new” ones is directed processes associated with new directions in breeding and the involvement of genetically different source material (Novoselskaya-Dragovich et al., 2007). Our data on the allele frequency of avenin-coding loci are in good agreement with this statement.

With the beginning of breeding work on oat in the Tyumen region, varieties appeared that possess a set of agronomically and adaptively significant characters for this region. This led to an increase in the frequency of certain alleles of avenin-coding loci, which can be considered markers of such genotypes or characters. At the same time, it caused a decrease in the frequency or even complete disappearance of alleles characteristic of foreign varieties. A2 and C2 alleles found in all groups of varieties, pro- bably, mark highly competitive gene associations that give their carriers important advantages in the natural climatic conditions of the region.

Monitoring changes in the genetic diversity of varieties over time allows judging the presence or absence of genetic erosion. In the works devoted to the assessment of genetic diversity in varieties of other crops, its values were 0.62–0.76 for soft wheat varieties created in Serbia and Italy (Novoselskaya-Dragovich et al., 2007), 0.5–0.6 in soft wheat varieties of Ukrainian selection (Zayka et al., 2014), and 0.42–0.64 in groups of durum wheat varieties originating from different countries of the world (Kudryavtsev et al., 2014). At the same time, a decrease in the value of this indicator in modern varieties is noted (Kudryavtsev et al., 2014; Lyalina et al., 2016).

The high values of genetic diversity identified as a result of our work and an increase in this indicator since 2000 indicate the absence of genetic erosion. It should be noted that, at different periods of time, the contribution of individual avenin-coding loci to the average gene diversity in varieties of oat in the region was not the same. In the period from 1970 to 2010, the Avn A and Avn B loci played an important role in the formation of genetic diversity. But currently, the maximum genetic diversity is observed at the Avn C locus. In our opinion, this suggests that the alleles of this locus may be important as markers of adaptively significant characters.

## Conclusion

The allele frequency of avenin-coding loci in varieties of common oat included in the State Register of Selection Achievements in the Tyumen region from 1929 to 2019 changed over time. The alleles characteristic of the varieties of foreign selection were replaced by “new” ones, specific to the varieties of local selection: Avn A4 (50.0 %), A2 (25.0 %), Avn B4 (50.0 %), Bnew6 (37.5 %), Avn C1 (37.5 %), C2 and C5 (25.0 %). These alleles are of great value as markers of agronomically and adaptively significant characters for the region in question.

Modern regionalized varieties of oat are characterized by high genetic diversity (0.75), which is associated with the use of heterogeneous source material in the breeding process. This allows obtaining varieties with high adaptive potentials in the climatic conditions of Western Siberia.

The high importance of genetic diversity in modern breeding varieties of the Scientific Research Institute of Agriculture of the Northern Trans-Urals and an increase in this indicator over the past 20 years indicate competently organized and effective breeding work with this crop in the Tyumen region.

## Conflict of interest

The authors declare no conflict of interest.
